# As time goes by: SMA neuromodulation and time perception while watching moving images with different editing styles. A tDCS study

**DOI:** 10.3389/fnhum.2025.1595599

**Published:** 2025-10-13

**Authors:** Alice Cancer, Stefania Balzarotti, Alessandro Antonietti, Adriano D’Aloia, Ruggero Eugeni

**Affiliations:** ^1^Department of Psychology, Università Cattolica del Sacro Cuore, Milan, Italy; ^2^Research Center in Communication Psychology, Università Cattolica del Sacro Cuore, Milan, Italy; ^3^Department of Letters, Philosophy, Communication, University of Bergamo, Bergamo, Italy; ^4^Department of Communication and Performing Arts, Università Cattolica del Sacro Cuore, Milan, Italy

**Keywords:** time perception, neuromodulation, tDCS, SMA, moving images, editing style, neurofilmology

## Abstract

Within the framework of a “neurofilmological” approach – which integrates film studies, cognitive psychology, and neuroscience – the present study explored how cinematographic editing influences the viewer’s perception of time. Previous behavioral research has shown that editing density affects temporal judgments. To investigate the neural mechanisms underlying this relationship, we examined the role of motor system activity, specifically the supplementary motor area (SMA), in time perception when individuals are exposed to moving images with different cinematographic editing styles. Forty-eight university students were assigned to one of three tDCS conditions (anodal, cathodal, or sham). They viewed nine silent video clips with different editing styles (master shot, slow-paced, fast-paced) that were specifically created for research. The participants rated perceived duration, time passage, action speed, and emotional engagement, while tDCS was applied for 20 min targeting the SMA. The results revealed that SMA excitability modulation affected duration estimates, time passage, and action speed judgments by interacting with the editing style of the clips. These findings highlight the importance of the SMA in modulating time perception during film viewing. Furthermore, they provide valuable insights into the neural mechanisms that shape the viewer’s perception of film time as an integral part of experiencing movement in cinema.

## 1 Introduction

The subjective experience of time remains a complex and elusive concept in cognitive psychology research and has been at the center of lively scientific debate in recent years (see Allman et al., 2014; Block and Gruber, 2014; Matthews and Meck, 2016; Thönes and Stocker, 2019). The mental representation of time is a multifaceted concept that encompasses processes such as temporal information processing (simultaneity, succession of events), the perception of temporal extension (duration estimation), and the subjective experience of time passage (the subjective feeling that time passes more quickly or slowly) (Thönes and Stocker, 2019). One of the most prominent models to account for time estimation is the Scalar Expectancy Theory (SET) (Gibbon et al., 1984), according to which time perception relies on an internal clock, in which a pacemaker generates pulses that are temporarily accumulated and stored in working memory. The accumulated pulses, which form the basis of time estimation, are then compared to a reference memory, which holds past experiences of accumulated pulses, allowing for a cognitive representation of time. A decision process ultimately determines the appropriate temporal response on the basis of this comparison.

The pacemaker-accumulator system is not merely a passive timer; rather, it actively contributes to behavioral state transitions (Killeen and Fetterman, 1988). Indeed, timing processing is the basis of anticipatory mechanisms and expectations of future occurrences. Humans, as well as animals, anticipate the occurrence of predictable future events through timing their own actions. From a behaviouralist perspective, the principle of anticipatory adaptation can be interpreted as an instance of temporal learning, as the sensitivity to the stable delay between the conditional and unconditional stimuli in Pavlovian conditioning is the mechanism that triggers the response (Ohyama et al., 2003). As a consequence, engaging in actions in response to temporal expectances, or even the mere mental representation of those actions, can influence the representation of time (Killeen and Fetterman, 1988)^1^.

Furthermore, the theoretical and empirical interaction between time experience and action has been explored in a multidisciplinary field of research that emphasizes the embodied nature of time perception (Altschuler and Sigrist, 2016; Coull et al., 2016; Meck and Ivry, 2016). Press et al. (2014) showed that the duration of a sensory stimulation (i.e., tactile vibration) was dilated by the concurrent action performance of a movement, compared with being at rest. Moreover, several studies have demonstrated that not only action execution, but also third-person observation of movements and actions can play a role in distorting or enhancing subjective time (Vatakis et al., 2014). Several findings have shown that a distortion of the perceived duration of visual stimuli can be induced by observed movement (for a review, see De Kock et al., 2021). An example of this is the subjective time dilation (Tomassini et al., 2011) that is induced by an object moving toward an observer compared with a static object (Van Wassenhove et al., 2008). Similarly, a greater density of events caused by greater stimulus velocity also leads to time dilation compared with a slower-moving stimulus (Kanai et al., 2006). Not only does actual movement affect time perception, but also implied or apparent movement was found to have a similar enhancing effect. Nather and Bueno (2011, 2012) reported that perceived durations of observations for pictures and sculptures representing implied body motions (i.e., stills of dance movements) were longer than those of stimuli representing unmoving figures. Even the exposure to abstract paintings that represented human motion was able to induce similar subjective time modulations (Nather et al., 2014). This lengthening effect has been attributed to different internal clock processes for moving versus static stimuli, driven by the recruitment of additional mechanisms linked to embodiment (e.g., procedural memory). In particular, movement observation induces temporal visuomotor representations on the basis of motor knowledge of human actions, which leads to internal clock acceleration (Nather and Bueno, 2011).

Finally, in an attempt to disentangle the contribution of a specific visuomotor mechanism relying on the motor representation of human actions from the confounding influence of perceptive biases in duration judgments of moving objects, Gavazzi et al. (2013) reported that the temporal estimation accuracy is improved by the correspondence between the stimulus kinematics and the observer’s motor competencies (i.e., participants were asked to replicate the duration of a dot moving in the vertical plane by moving their right arm along the vertical plane). These results suggest that the temporal mechanism of visual motion relies on a temporal visuomotor representation shaped by motor knowledge of human actions. This interpretation is consistent with the consolidated role of the mirror neuron system in action observation (Gallese et al., 1996; Rizzolatti et al., 1996), which supports the notion that the motor brain areas responsible for the execution of a specific action are activated during observation of the same action performed by another individual.

Neuroscientific research has confirmed that the motor system is involved in time perception processes (Macar and Vidal, 2004). Although temporal perception and estimation tasks involve a distributed brain network including cortical and ventral structures (e.g., the basal ganglia, the cerebellum, premotor, parietal and dorsolateral prefrontal cortices) (Meck, 2006; Paton and Buonomano, 2018; Nani et al., 2019), neuroimaging studies have highlighted a key role of the Supplementary Motor Area (SMA) in temporal processing (Coull, 2004; Macar and Vidal, 2004; Macar et al., 2006; Wiener et al., 2010; Coull et al., 2011, 2016; Schwartze et al., 2012; Nani et al., 2019; Teghil et al., 2019; Capizzi et al., 2023). Specifically, the activation of this area, which is typically involved in motor control and planning (Tanji, 2001; Nachev et al., 2008), is proportional to the estimated duration of the visually presented temporal stimulus (Coull et al., 2015), regardless of its association with a motor response planning task. Further evidence on the role of the SMA in temporal processing tasks can be found in the neuroscience literature. It has been observed that temporal ability is impaired in patients with SMA lesions (Halsband et al., 1993). Electrophysiological studies based on event-related potentials (ERPs) have recorded an increase in SMA activation proportional to the estimated temporal duration. Specifically, variations in amplitude (Wiener et al., 2012) and latency (Ng et al., 2011) of the Contingent Negative Variation (CNV) component have been observed as a function of the presented stimulus duration, with an activation profile indicating anticipation of the stimulus end (Mento et al., 2013), thus enabling temporal decision making. Additionally, Kononowicz and Rijn (2015) observed an increase in the beta oscillatory rhythm detected in the SMA, which was proportional to the estimated duration of the interval produced in a temporal interval reproduction task (Wiener et al., 2018). The increase in SMA activation as a function of subjective duration was interpreted by Coull et al. (2015) as confirmation of the preferential role of this area in the process of accumulation, a key component of temporal perception in the internal clock model (Gibbon et al., 1984). Through an fMRI study, Wencil et al. (2010) confirmed Coull and colleagues’ hypothesis by identifying a neurofunctional basis for the accumulator in a network that includes the SMA. To further confirm the involvement of the SMA in time duration perception, a study conducted by Herrmann et al. (2014) demonstrated that greater preSMA activation was observed in participants who exhibited greater resistance to the temporal illusion phenomenon. These participants were more accurate in an auditory stimulus temporal discrimination task, regardless of the presence or absence of the illusion.

Non-invasive brain stimulation (NIBS) techniques, such as transcranial magnetic (TMS) and transcranial electric stimulation (tES), have been extensively used to investigate the neural basis of time perception (for a review, see Mioni et al., 2020). Nonetheless, compared with neuroimaging research, brain stimulation studies have thus far failed to consistently support a key role of the SMA in the processing of temporal information (Wiener, 2014; Mioni et al., 2020; Capizzi et al., 2023). On the one hand, TMS studies (e.g., Dusek et al., 2011; Giovannelli et al., 2014) have revealed weak or null effects of SMA stimulation on participants’ behavioral performance at time perception tasks (e.g., accuracy of duration estimates). On the other, a few studies employing transcranial Random Noise Stimulation (tRNS) to perturb SMA functioning in explicit timing tasks (Wiener et al., 2018; Capizzi et al., 2023) have reported evidence of an induced overestimation of durations. Overall, these mixed results leave space to further explore the potential causal role of SMA activity in time perception.

The crucial role of action processing in the subjective time experience opens the possibility of exploring this mechanism within the cinematographic context – not simply considering audiovisual as a substitute for natural stimuli, but taking into account their specific linguistic and semiotic aspects (D’Aloia and Eugeni, 2014; Gallese and Guerra, 2015, 2022; D’Aloia, 2021). Indeed, action representation finds one of its richest expressions in cinema and editing can be used to control the temporal unfolding of actions depicted in a film (Bordwell, 2013). Previous research has explored the neural mechanisms underlying watching films (Hasson et al., 2008; Heimann et al., 2014) and the impact of various editing techniques on viewers’ temporal perception (Wied et al., 1992; Cohen et al., 2017). To date, however, only a few studies have examined how editing techniques influence the perceived duration of a scene – whether extended or compressed – compared with their actual duration. Editing techniques are typically used by filmmakers in deliberate attempts to manipulate a scene’s perceived duration, given the inherent contrast between actual screen time and narrative time. For example, elliptical editing is used to compress time by omitting parts of an action while maintaining continuity, whereas overlapping editing is used to extend time by repeating action from different angles (Bordwell, 2013). Notably, editing can be considered as one of the forms of movement that characterize cinematographic images (Shimamura et al., 2014; Sobchack, 2016; Heimann et al., 2017).

The first experimental attempt to investigate how editing techniques influence viewers’ perception of duration in suspense scenes was conducted by de Wied et al. (1992), who reported that suspense scenes were perceived as lasting longer when preceded by introductory scenes with higher degrees of compression, thus suggesting that a fast-paced succession of cuts enhanced the sense of extended duration. More recently, Eugeni et al. (2020) and Balzarotti et al. (2021) investigated how editing density (that is, the number of shots – and hence of cuts and transitions between them – in an audiovisual segment in relation to its duration) influences viewers’ perception of time. In their experiment, the participants watched silent video clips that represented different types of routine actions (e.g., drinking water, slicing bread, and moving object on a table) and that were edited using varying editing density (fast-paced, slow-paced, and unedited). The participants were then asked to report duration judgments and subjective time experience for each clip. The results revealed that – compared to unedited clips – fast-paced clips led to perceive time as if it passed more quickly but, at the same time, participants tended to overestimate the duration of the clips (Balzarotti et al., 2021). Moreover, the type of action represented in the clip also influenced participants’ judgments. Duration judgments were more accurate when the actor performed an action characterized by linearity (i.e., repetitions or iterations) and by a sequence of sub-actions clearly oriented toward a goal (e.g., grasping, lifting, holding a glass and bringing it to mouth to drink water; Eugeni et al., 2020).

Similar results were reported by Kovarski et al. (2022), whose findings revealed that edited scenes – either maintaining spatiotemporal continuity or introducing discontinuity in time, space, and action – were perceived as longer than scenes with no editing. Notably, while Balzarotti et al. (2021) used silent video clips that were created *ad hoc* with a duration on the order of tenths of seconds (11000–13500 ms), Kovarski et al. (2022) employed shorter clips (2500–3500 ms) that consisted in excepts from a movie. Also, the number of cuts was different (10–12 cuts in the former study, a single cut in the latter). Finally, a recent study by Liapi et al. (2024) examined the perceived durations of videos that represented various actions (e.g., to drink a cup of tea) and were manipulated via three editing techniques: expanded (5 cuts), compressed (3 cuts), and real-time (1 cut). The results revealed that expanded scenes were perceived as significantly longer than both compressed scenes and real-time scenes, whereas real-time scenes were also estimated to last longer than compressed ones. However, the number of cuts in the scene was not the only factor influencing perceived duration, since duration estimates also differed according to the type of action represented in the clip. In addition, the authors speculated that other factors influencing participants’ duration perception may have been the varying actual durations of scenes and differences in attentional saliency due to motion, color, and intensity in each scene (Liapi et al., 2024).

### 1.1 The present study

On the basis of previous evidence that the cinematographic editing style affects temporal judgments (Eugeni et al., 2020; Balzarotti et al., 2021), the present study aimed to investigate the neural mechanisms underlying this relationship. Specifically, we aimed to study the role of the motor system in time perception during exposure to video clips depicting actions with varying editing densities by modulating the excitability of the SMA using transcranial direct current stimulation (tDCS).

Concerning editing, following previous studies, we examined whether cinematographic editing density influences viewers’ perception of time. On the basis of the results reported by Balzarotti et al. (2021), we expected that the number of cuts would affect both duration estimates and subjective time passage: A faster pace of editing (i.e., a higher number of cuts) should lead participants to perceive time as flowing faster, but also to overestimate the duration of the video clips.

Since it has been successfully used to modulate the excitability of the SMA in numerous previous studies (e.g., Carlsen et al., 2015; Hupfeld et al., 2017; Nomura and Kirimoto, 2018), tDCS was selected as the optimal technique for our investigation. More precisely, we hypothesized that enhancing SMA excitability would strengthen the link between motor knowledge and visual motion perception, thereby compensating for subjective time dilation effects and ultimately leading to more accurate duration perception. Conversely, for the same reasons, we hypothesized that decreased excitability would lead to greater susceptibility to movement-related time dilation biases, resulting in distorted duration perception. Furthermore, we hypothesized that the experience of time passage would vary across stimulation conditions due to differences in duration perception.

## 2 Materials and methods

### 2.1 Participants

Forty-eight undergraduate students aged 21–31 years (M_*age*_ = 25.1; SD = 2.41; *F* = 62.5%) volunteered to participate in the study. Students were recruited via email invitations and advertisements on social media platforms. The participants were assigned to one of three experimental conditions (i.e., anodal, cathodal, or sham), by stratifying by sex (χ^2^ = 0.53; ns) and university level (χ^2^ = 5.00; ns).

### 2.2 Transcranial direct current stimulation (tDCS)

A direct current of 1.5 mA intensity was generated by a battery-driven stimulator (BrainStim - E.M.S., Bologna, Italy) and delivered for 20 min through two rubber electrodes, inserted into saline-soaked sponges covered with conductive gel. A 5 × 5 cm^2^ stimulation electrode (either anode or cathode, current density of 0.06 mA/cm^2^) was placed 1.8 cm anterior to the measured location of Cz (based on the international 10-20 system for EEG electrode placement), according to Carlsen et al. (2015). Both the stimulation duration (20 min) and current intensity (1.5 mA) fall within the range of parameters commonly used in previous tDCS studies targeting the SMA (e.g., Hsu et al., 2011; Kaminski et al., 2013; Vollmann et al., 2013; Bolzoni et al., 2015; Nomura and Kirimoto, 2018; Sato et al., 2024). A 7 × 5 cm^2^ reference electrode was placed over the right upper arm. An extra-cephalic montage was chosen to minimize potential confounding effects in the brain that could arise from the placement of the reference electrode. In the control (sham) condition, participants received 1.5 mA of current to give the impression of stimulation, but the current ramped down to 0 mA after a few seconds.

### 2.3 Stimuli

All video stimuli used in the experiment are openly available at https://www.ritmi.net/2025/09/07/seemit/. We employed the same experimental stimuli created and used in Eugeni et al. (2020) and Balzarotti et al. (2021). Since the details regarding video editing are reported in these studies, we provide a brief description here. Nine video clips representing different action types performed by a male actor were shot in a professional studio by an experienced videography crew using two sets of seven cameras. Video production included nine different shot sizes and angles.

The video clips were edited according to three cinematographic editing styles: (a) in the master shot (no editing) actions were shown from a frontal perspective, a medium shot, with no cuts; (b) the slow-paced editing included four “match-on-action” cuts, following the rules of continuity editing; (c) fast-paced editing included a greater number (10–12) of cuts and a greater variety of angle/distance changes (e.g., point-of-view shots, plongées, close-ups, cut-in shots) than slow-paced editing and was intended to imitate the so-called intensified continuity editing without violating continuity rules. In addition, the actor was asked to perform three different goal-directed routine actions (each action was edited according to the editing styles described above). In more detail, the male actor was instructed (1) to pour water into a glass and drink it (“drinking water”); (2) to cut a loaf of bread using a knife (“cutting bread”); (3) to change the position of a loaf of bread and an empty glass on a table (“moving objects”). Notably, duration was maintained constant across the different action types. In other words, although differently edited, the three videos of the same action had the same duration (13.5 s for “drinking water,” 11 s for “cutting bread,” and 11 s for “moving objects”).

### 2.4 Procedure

Written informed consent was obtained from the participants prior to recruitment. After providing demographic information (e.g., age, gender), the participants were randomly assigned to one of three experimental conditions: (1) anodal tDCS over the SMA, (2) cathodal tDCS over the SMA, or (3) sham tDCS. Each participant underwent a single experimental session. Five minutes after the beginning of the stimulation, the participants were presented with the 9 video clips following Balzarotti and colleagues’ procedure (Balzarotti et al., 2021). The order of the clips’ presentations was counterbalanced. After each video clip, the participants were asked to rate (1) the perceived duration of the clip by indicating a numerical value between 1 and 30 s; (2) the subjective passage of time on a 9-point scale (1 = “time dragged”; 9 = “time flew”); (3) the action speed on a 9-point scale (1 = “very slow”; 9 = “very fast”). Furthermore, the participants were asked to rate their interest, emotional engagement, and boredom on a 7-point scale (1 = “not at all”; 7 = “very much”).

The experimental task was built using PsychoPy v.3.1.0 (Peirce et al., 2019), which was used for both stimulus presentation and response recording. The study was approved by the Ethics Committee of the Università Cattolica del Sacro Cuore of Milan, Italy (approval code: 161-24) according to the standards of the Helsinki Declaration (World Medical Association, 2001).

### 2.5 Analyses

The sample size (*n* = 48) was calculated to achieve a statistical power of 0.9 for a mixed-design ANOVA (3 × 3), assuming an effect size of 0.25 (Cohen’s f) and a significance level (α) set at 0.05. To explore the effects of tDCS, editing style, and action type on time processing (i.e., duration accuracy – calculated as the duration estimate divided by the actual clip duration, time passage, and action speed) and emotional involvement (i.e., engagement, interest, and boredom), mixed factorial ANOVAs (3 × 3 × 3) with Bonferroni pairwise comparisons were used. The partial eta squared (η_*p*_^2^) is reported as the effect size, as it is more appropriate for factorial designs, isolating each factor’s unique contribution to explained variance (Richardson, 2011). Analyses were performed by using the software Jamovi (version 2.6) (The jamovi project, 2025).

## 3 Results

### 3.1 Duration estimates

**FIGURE 1:**
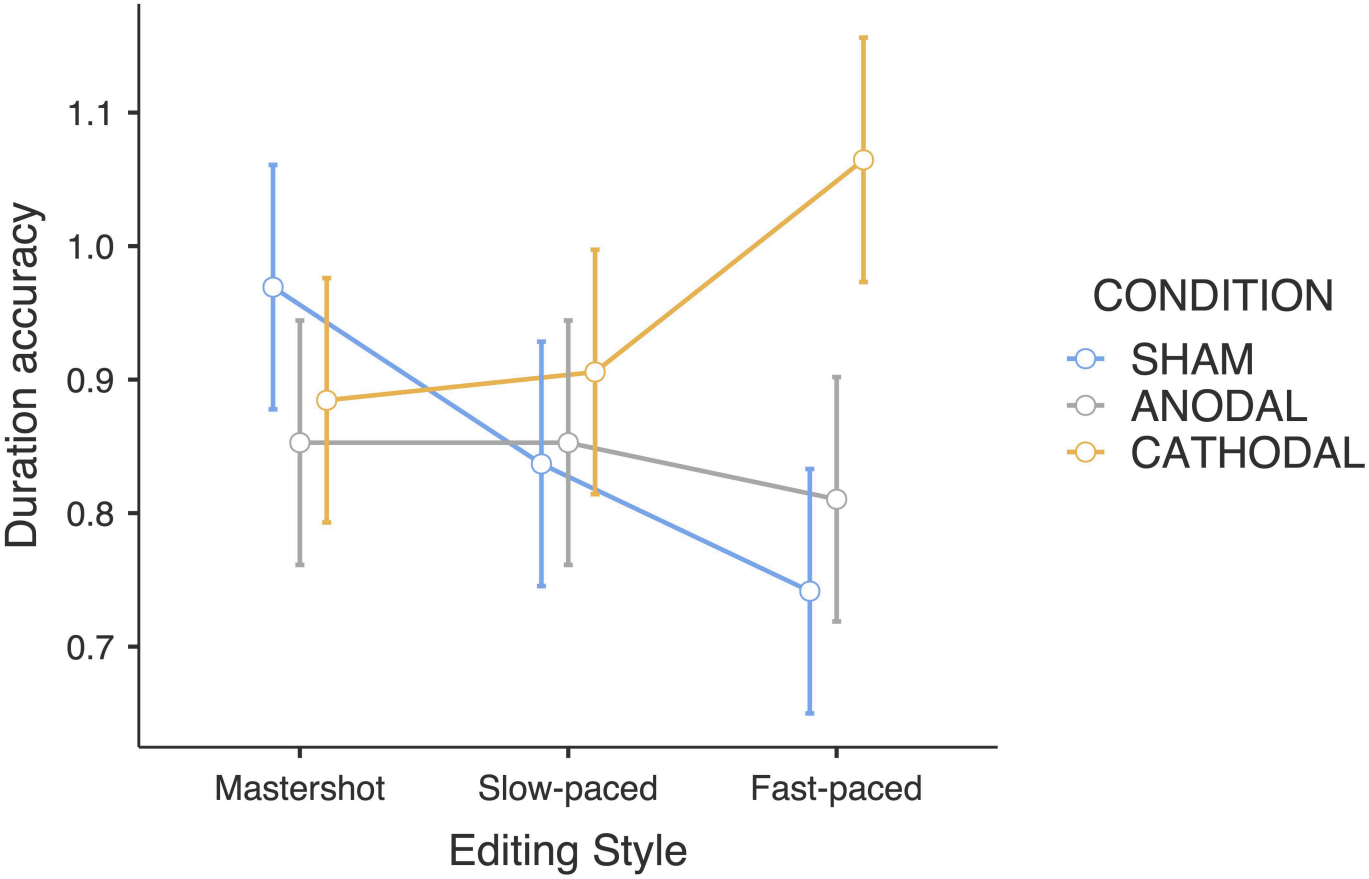
Accuracy of duration estimates of the “moving object” clips (Editing style × tDCS Condition).

The interaction between editing style and tDCS condition yielded a significant effect on duration estimates (*F*_4_,_180_ = 2.74; *p* < 0.05; η_*p*_^2^ = 0.11). Bonferroni pairwise comparison revealed that the duration of fast-paced edited clips was estimated to be longer compared to the master shot (*p* < 0.05) in the cathodal condition. The main effect of action type was also significant (*F*_2_,_180_ = 9.45; *p* < 0.001; η_*p*_^2^ = 0.17) with “drinking water” clips generating shorter duration estimates than “cutting bread” (*p* < 0.05) and “moving objects” clips (*p* < 0.001) (Table 1). *Post hoc* analyses (i.e., considering each action type separately) revealed that the interaction effect between tDCS and editing style was mainly driven by the “moving objects” clips, in which a clear-cut effect that confirmed the initial hypothesis was found (*F*_4_,_90_ = 3.51; *p* < 0.05; η_*p*_^2^ = 0.13). The participants who received the sham stimulation reported shorter duration estimates for the “moving objects” clips, which was consistent with the increasing direction of the editing speed: in other words, the faster the editing style was, the shorter the duration estimates (uncorrected *p* < 0.01). Conversely, the opposite trend was observed in the cathodal group: the faster the editing style was, the longer the duration estimates. Finally, participants who received anodal stimulation did not change their duration estimates according to the editing style (Figure 1).

**TABLE 1:** Duration accuracy estimates (Editing style × tDCS Condition × Action type).

Action type	tDCS	Editing style	Mean	SD
Drinking water	sham	Master shot	0.671	0.450
		Slow-paced	0.653	0.273
		Fast-paced	0.699	0.285
	anodal	Master shot	0.722	0.298
		Slow-paced	0.750	0.243
		Fast-paced	0.796	0.359
	cathodal	Master shot	0.755	0.380
		Slow-paced	0.870	0.298
		Fast-paced	0.926	0.304
Cutting bread	sham	Master shot	0.699	0.204
		Slow-paced	0.813	0.440
		Fast-paced	0.847	0.537
	anodal	Master shot	0.790	0.300
		Slow-paced	0.841	0.287
		Fast-paced	0.790	0.283
	cathodal	Master shot	0.841	0.400
		Slow-paced	0.864	0.304
		Fast-paced	1.057	0.386
Moving objects	sham	Master shot	0.969	0.517
		Slow-paced	0.837	0.461
		Fast-paced	0.742	0.260
	anodal	Master shot	0.853	0.265
		Slow-paced	0.853	0.248
		Fast-paced	0.810	0.327
	cathodal	Master shot	0.885	0.340
		Slow-paced	0.906	0.347
		Fast-paced	1.065	0.429

### 3.2 Time passage and action speed Judgments

**FIGURE 2:**
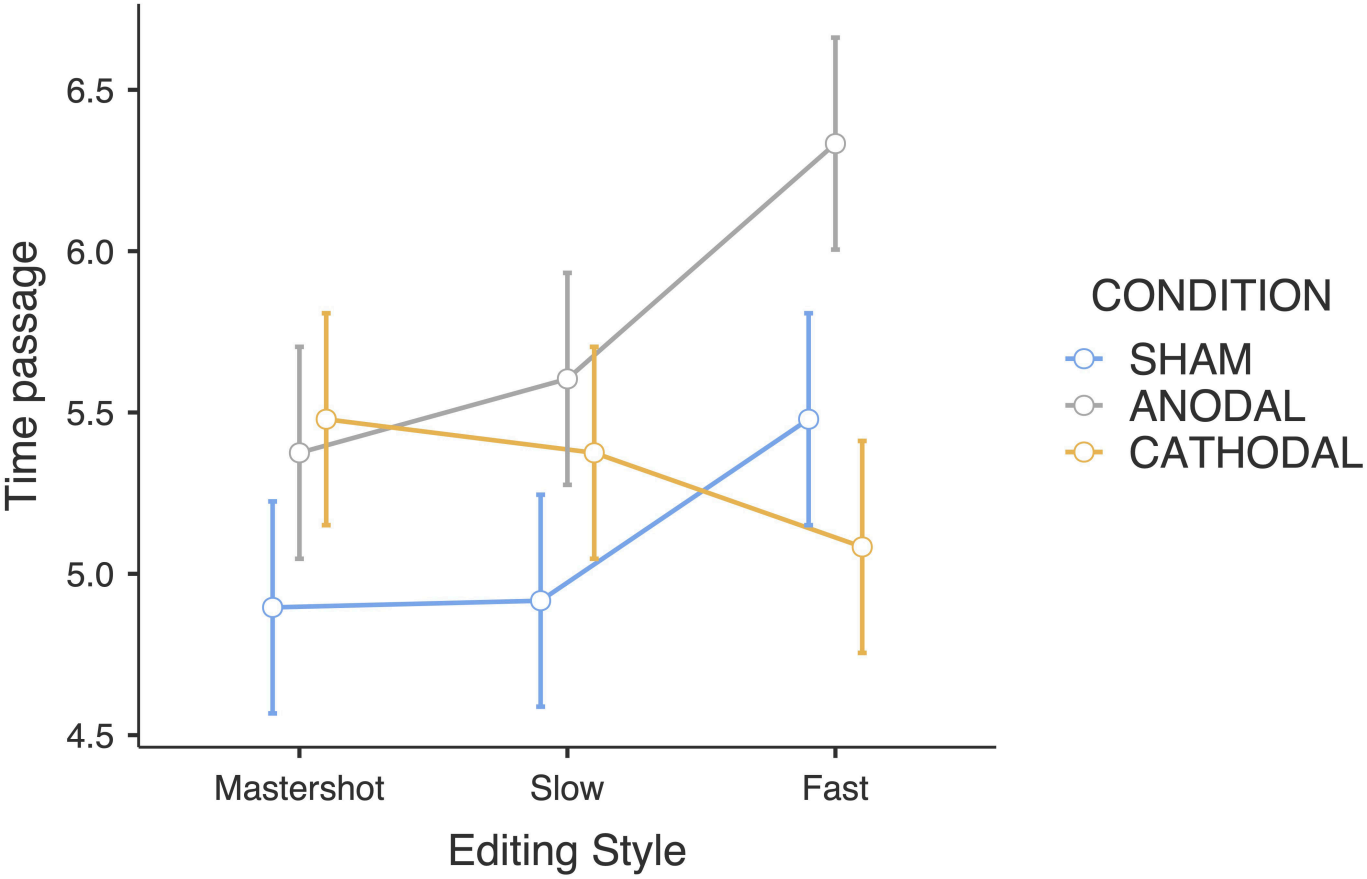
Time passage judgments estimated marginal means (Editing style × tDCS Condition).

Similar to the duration judgments, a significant interaction effect between tDCS and editing style was found (*F*_4_,_180_ = 2.77; *p* < 0.05; η_*p*_^2^ = 0.11). *Post hoc* pairwise comparisons revealed that participants who received anodal stimulation reported that fast-paced edited clips elapsed faster than both slow-paced (*p* < 0.05) and master shot clips did (*p* < 0.01). Therefore, in this group, the faster the editing style of the clips is, the higher the time passage ratings. Moreover, participants in the anodal group rated the subjective time to pass faster when watching the fast-paced clips than did participants in the cathodal group (*p* < 0.01). The estimated marginal means revealed that the participants who received cathodal stimulation judged that time elapsed slower when the editing style was faster, indicating an opposite trend compared with the anodal group (Figure 2). Finally, the sham group showed a trend similar to that of the anodal group, even though there was lower rating variability between the editing styles. Notably, the aforementioned statistically significant pairwise differences were computed without correcting for multiple comparisons.

**FIGURE 3:**
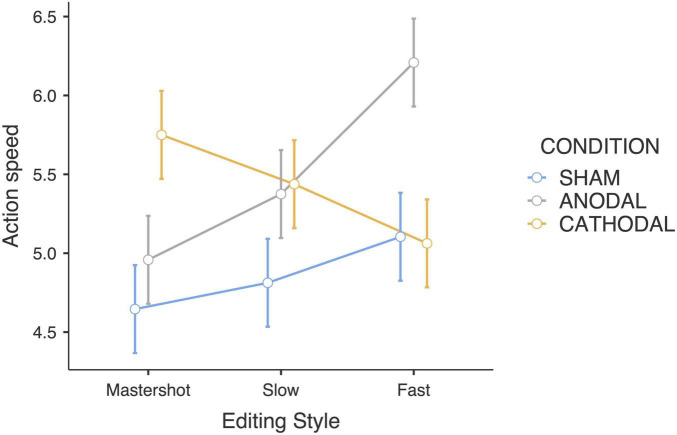
Action speed judgments estimated marginal means (Editing style × tDCS Condition).

Consistent with the time passage judgments, the interaction between the tDCS condition and editing style yielded a significant difference in the action speed judgments (*F*_4_,_180_ = 6.58; *p* < 0.001; η_p_^2^ = 0.23). In the anodal group, the actions in fast-paced edited clips were rated as faster than those in the master shot clips were (*p* < 0.001), as shown by Bonferroni pairwise comparisons. In contrast, we observed the opposite trend in the cathodal group: fast editing led to a slower action speed (Figure 3). Compared with the anodal group, the sham stimulation group presented slightly increased ratings along with the pace of the editing, although with lower variability.

### 3.3 Emotional involvement

The editing style, but not tDCS or action type, affected participants’ emotional involvement. A significant main effect of the editing style emerged on the engagement ratings (*F*_2_,_180_ = 4.55; *p* < 0.05; η_p_^2^ = 0.09). Bonferroni pairwise comparisons revealed that fast-paced edited clips were rated as more engaging than master shot clips were (*p* < 0.05). Similarly, the analysis yielded a significant main effect of editing style on interest (F_2;180_ = 8.16; *p* < 0.001; η_p_^2^ = 0.15), with fast-paced edited clips rated as more interesting than the master shot clips (*p* < 0.001), as shown by the pairwise comparisons. Finally, boredom was not affected by any of the independent variables.

## 4 Discussion

The modulation of SMA excitability affected the objective (i.e., duration estimates) and subjective (i.e., time passage and action speed judgments) measures of time perception, by interacting with the editing style of the clips.

Consistent with our initial hypothesis and with neuroscientific evidence showing SMA involvement in accurate temporal processing (e.g., Coull et al., 2015), increased excitability of the SMA induced duration perceptions that were not susceptible to editing style influences. Participants who received anodal stimulation over the SMA did not adjust their duration estimates according to the editing style, but instead reported consistent estimates, regardless of the editing density. This result is in line with the fMRI findings by Herrmann et al. (2014), who reported that SMA activation was a predictor of individual differences in temporal-change sensitivity, with reductions in susceptibility to illusory distortions. We suggest that increased SMA excitability facilitates the development of a temporal visuomotor representation shaped by motor knowledge of human actions, which ensures a more precise match between the internal models of action and the visual kinematics of the observed motion. This, as a result, improved the temporal mechanism of visual motion, compensating for temporal sensory limitations caused by subjective time dilation effects. In contrast, decreased neuronal excitability of the SMA through cathodal stimulation yielded duration estimates directly influenced by movement density; specifically, the duration of fast-paced edited clips was estimated to be the longest, whereas that of the master shot clips was estimated to be the shortest. This finding is consistent with the subjective time dilation induced by visual stimulus velocity (Kanai et al., 2006; Tomassini et al., 2011). We argue that such bias cannot be effectively modulated by exploiting sensorimotor representations due to the inhibition of SMA involvement.

Consistent with previous findings (Eugeni et al., 2020; Liapi et al., 2024), duration estimates were also influenced by the type of action represented in the clips. More precisely, the three actions differed in terms of intentionality and goal orientation and, therefore, in terms of predictability. The clearest the intentionality of the action, as in “drinking water” clips, the easiest is the anticipation of the action’s ending. In contrast, the endings of actions with an undefined global intention (i.e., the “moving objects” clips) are the most difficult to anticipate. Our results, consistent with Eugeni and colleagues’ findings (2020), showed that the clarity of intentionality influenced the duration estimates: more predictable actions were estimated to be shorter than less predictable ones. Indeed, previous evidence has shown that individuals tend to perceive the onset of predictable movements as delayed while anticipating their consequences, leading to an underestimation of their duration (Haggard et al., 2002). The interaction effect between neuromodulation and editing style was found to be stronger in the “moving objects” clips. We argue that the non-predictability of this action assured a duration measure that was free of potential anticipatory biases.

With respect to the subjective experience of time passage, the modulation of SMA excitability had the opposite effect on the time passage and action speed judgments of clips with different editing styles as a function of neuromodulation polarity (i.e., anodal vs. cathodal). More precisely, anodal stimulation amplified the effect of editing speed on temporal subjective judgments, in the direction of faster time passage and faster action perceptions along with faster editing pace. This effect is consistent with the findings of Balzarotti et al. (2021), who reported that an increased number of cuts in a scene influences time judgments, accelerating the perceived flow of time. Likewise, previous results from Wearden (2005) showed that participants perceived time as passing more quickly while watching an action film with a fast editing style than when watching a relaxation film. The direction of this effect was reversed by cathodal stimulation: The time passage and action flow were perceived to elapse more slowly in faster-paced edited clips. While earlier studies (Droit-Volet and Wearden, 2016; Droit-Volet et al., 2017) suggested that judgments of time passage are unrelated to duration perception, more recent evidence (Martinelli and Droit-Volet, 2022) indicates that the perceived speed of time increases as stimulus duration decreases, which aligns with our results.

It bears noting, however, that not all our results concerning duration estimates are consistent with previous research and hypotheses. First, compared with the results reported by Eugeni et al. (2020) and Balzarotti et al. (2021), we were unable to replicate a significant main effect of editing density on duration estimates, and thus the hypothesis regarding the influence of editing density on duration overestimation was overall only partially confirmed. In more detail, concerning the “moving object” action type, the participants in the sham group reported shorter (rather than longer) durations for fast-paced video clips than for unedited and slow-paced clips. Second, although the analyses revealed a significant effect of anodal vs. sham stimulation on duration estimates, overall, significant differences mainly and more consistently concerned the cathodal group – with disruption of SMA excitability leading to longer duration estimates and to a subjective experience of time dragging. Inconsistencies in the results of research using brain stimulation to assess the role of the SMA in time perception have been previously reported (e.g., null or weak effects), and a possible explanation is that they may be due to the existence of functionally distinct areas in the SMA (e.g., preSMA; Mioni et al., 2020).

This study is the first to explore the neural basis of time perception in relation to movie clips with different editing styles. Our findings highlighted the role of the SMA in modulating time perception during film viewing, showing that increased involvement of the sensorimotor system produces more accurate duration estimates, whereas its inhibition enhances susceptibility to editing-induced distortions. Additionally, the predictability of actions influences time perception, with clearer intentionality leading to shorter perceived durations. This investigation offers an important contribution by bridging neuroscience and film studies, shedding light on how embodied mechanisms underpin the perception of cinematographic time. In particular, these conclusions open a broader discussion about the specificity of the film viewing experience compared with the ordinary one. In fact, it can be assumed that the cinematographic experience is profoundly shaped by the multiplicity of stimuli of moving objects and subjects: the actors engaged in the actions, the camera, and the editing itself perceived as a form of shifting the point of view. The need to coordinate these different flows into a coherent pattern produces the temporal nature of the audiovisual viewing experience, also through a specific and peculiar involvement of the SMA.

### 4.1 Limitations and future directions

Our study is not without its limitations. First, although we report that duration estimates were influenced not only by editing density and changes in SMA excitability, but also by the type of action represented in the clips, future studies should investigate a wider range of actions to further disentangle the interplay between sensorimotor representations and temporal processing. Expanding this line of research could provide a more comprehensive understanding of the factors (e.g., linearity, intentionality, repetition) shaping time perception in dynamic visual contexts.

Second, as already noted by Balzarotti et al. (2021), although the video clips employed in this study were created trying to maintain editing density as the main and most prominent variation, we cannot exclude that other derivative modifications (e.g., amount of information on the screen, image motion, angle of shots) could be a confounding driving factor behind our results.

Third, although we aimed to isolate the effects of tDCS, potential confounding factors, such as individual variability in responsiveness to brain stimulation or interactions with cognitive state, cannot be completely ruled out.

Finally, in this study we focused on the neural dynamics of time perception during film viewing, and in particular on the possible role of the SMA. Nonetheless, future work comparing our findings with computational models could help to further clarify the evidence obtained – specifically, the suggestion that the SMA may function as a neural hub supporting the dynamic updating of temporal predictions, thereby shaping the subjective experience of how “time passes” during audiovisual experience.

## Data Availability

The raw data supporting the conclusions of this article will be made available by the authors, without undue reservation.

## Footnotes

1 Many scholars have sought to account for the mechanisms by which motor and perceptual systems integrate information over time through formal models of the mind inspired by probabilistic prediction algorithms – most notably Bayesian inference updating, Kalman filtering, and Hidden Markov models (Basgol et al., 2022; Glasauer and Shi, 2022; Mugruza-Vassallo et al., 2022; Sadibolova and Terhune, 2022; Rhodes et al., 2024). Within these frameworks, time perception is typically formalized as a process of iterative state updating, in which internal temporal estimates are continuously adjusted in response to prediction errors and contextual cues. Kalman filtering, in particular, has been advanced as a prominent account of sensorimotor timing (Knelange and López-Moliner, 2019; Jörges and Harris, 2024; the latter uses stimuli in a VR environment). Likewise, Hidden Markov approaches have been employed to describe neural state transitions during movie viewing (Meer et al., 2020). In this study, our primary objective is to examine the role of the supplementary motor area (SMA) in shaping subjective perception during naturalistic film viewing. Accordingly, our focus lies on neural dynamics rather than on the implementation of a formal, computational model of temporal estimation.
